# Low Computational Cost for Sample Entropy

**DOI:** 10.3390/e20010061

**Published:** 2018-01-13

**Authors:** George Manis, Md Aktaruzzaman, Roberto Sassi

**Affiliations:** 1 Department of Computer Science and Engineering, University of Ioannina, Ioannina 45110, Greece; 2 Department of Computer Science and Engineering, Islamic University Kushtia, Kushtia 7003, Bangladesh; 3 Dipartimento di Informatica, Università degli Studi di Milano, Crema 26013, Italy

**Keywords:** Sample Entropy, algorithm, fast computation, kd-trees, bucket-assisted algorithm

## Abstract

Sample Entropy is the most popular definition of entropy and is widely used as a measure of the regularity/complexity of a time series. On the other hand, it is a computationally expensive method which may require a large amount of time when used in long series or with a large number of signals. The computationally intensive part is the similarity check between points in *m* dimensional space. In this paper, we propose new algorithms or extend already proposed ones, aiming to compute Sample Entropy quickly. All algorithms return exactly the same value for Sample Entropy, and no approximation techniques are used. We compare and evaluate them using cardiac inter-beat (*RR*) time series. We investigate three algorithms. The first one is an extension of the kd-trees algorithm, customized for Sample Entropy. The second one is an extension of an algorithm initially proposed for Approximate Entropy, again customized for Sample Entropy, but also improved to present even faster results. The last one is a completely new algorithm, presenting the fastest execution times for specific values of *m*, *r*, time series length, and signal characteristics. These algorithms are compared with the straightforward implementation, directly resulting from the definition of Sample Entropy, in order to give a clear image of the speedups achieved. All algorithms assume the classical approach to the metric, in which the maximum norm is used. The key idea of the two last suggested algorithms is to avoid unnecessary comparisons by detecting them early. We use the term *unnecessary* to refer to those comparisons for which we know a priori that they will fail at the similarity check. The number of avoided comparisons is proved to be very large, resulting in an analogous large reduction of execution time, making them the fastest algorithms available today for the computation of Sample Entropy.

## 1. Introduction

The use of conditional entropy to measure the regularity (or complexity) of time series or signals has become quite popular. The two most commonly used measures of entropy are Approximate Entropy (ApEn) and Sample Entropy (SampEn), which have been used extensively in biological signals analysis over the last 20 years [1].

Approximate Entropy was first proposed by Pincus [2] as a measure of systems complexity. It quantifies the unpredictability of fluctuations in a time series; the *approximate* part of its name came from the fact that the index was derived from the estimate of Kolmogorov–Sinai [3,4] entropy—a theoretical metric employed in the context of nonlinear dynamical systems. Many potential applications [5,6,7,8,9,10] of this metric for biological signals analysis are found in the literature.

To date, hundreds of published papers have employed ApEn, first praising its quality but also, over the years, evidencing its limits. A related index, Sample Entropy (SampEn), was introduced by Richman and Moorman [11], and is actually a slightly different way to compute the metric. SampEn attempts to improve ApEn, being a less biased metric for the complexity of the system (at the price of a larger variance of the estimates). This is obtained by evaluating the conditional Rényi entropy of order 2, instead of the classical conditional entropy. Like ApEn, SampEn has also been used in various scientific fields, such as neonatal heart rate signals analysis [12], effects of mobile phones radiation on heart rate variability (HRV) [13], sleep apnea detection [14], epilepsy detection from electroencephalogram (EEG) signals [15], detection of atrial fibrillation [16], in the analysis of human postural data [17], etc.

The computation of each metric requires checking the similarity of small patterns (or templates of size *m*), constructed from the series. The number of similarity checking, which is the most computationally intensive part of their computations, increases quadratically when increasing the series length *N*. The proposed study provides powerful algorithms which might be helpful for the usage of SampEn in real-time applications from the computational point of view. Earlier stages of this work have been presented in [18] and [19], where Approximate Entropy was investigated. This paper focuses on Sample Entropy. The contribution of the paper can be summarized in the following points:

- an improvement to the kd-algorithm used by other researchers [20,21] for the fast computation of Sample Entropy is introduced
- an algorithm computing Sample Entropy quickly is proposed, which is an extension to the bucket-assisted algorithm [19] initially introduced for Approximate Entropy. This algorithm has been customized to compute Sample Entropy, and has also been extended to present even faster execution times by sorting the points inside the buckets and by tuning the size of the buckets
- a completely new algorithm is presented which is faster than any other algorithm when used for specific values of *m*, *r*, and signal lengths
- finally, a comparison of all algorithms is presented, based on experimental results collected using implementations of the algorithms in C programming language. The implementation in C allows the programmer to optimize the code in a relatively low level, without heavy software layers lying between the programmer and the hardware.

This paper assumes the classical definition of Sample Entropy, in which the maximum norm is used as a distance between the vectors. Algorithms 1 and 4 can be easily modified to support some other norms, instead of the maximum one. Algorithms 2 and 3 are not appropriate for other norms. However, we must note that in almost all applications of Sample Entropy, the maximum norm is used as the distance between two vectors.

## 2. Sample Entropy

Suppose a time series with *N* points is given:

(1) $$x=x_{1},x_{2},\cdots ,x_{N},$$

from which a new series $\vec{x}$ of vectors of size *m* is constructed. Sometimes this series is also referred to in the literature as *pattern* or *template*:

(2) $$\vec{x}={\vec{x}}_{1},{\vec{x}}_{2},\cdots ,{\vec{x}}_{N-m+1},\;\;\;\;\;\;\;{\vec{x}}_{i}=\left(x_{i},x_{i+1},\cdots ,x_{i+m-1}\right).$$

The two vectors ${\vec{x}}_{i}$ and ${\vec{x}}_{j}$ are considered similar if the maximum distance between all of their corresponding elements is within a selected threshold *r*. This threshold is also termed the *tolerance of mismatch* between two vectors; i.e.,:

(3) $$|x_{i+k}-x_{j+k}|\leq r,\;\;\;\forall \left\{i,j\right\},\;\;\;0\leq k\leq m-1.$$

In the following, the notation ${\left|\left|{\vec{x}}_{i}-{\vec{x}}_{j}\right|\right|}_{m}\leq r$ will be used to express the similarity of two vectors of size *m*. Given the distance *r*, the number of vectors of length *m* similar to ${\vec{x}}_{i}$ are given by $n_{i}^{m}\left(r\right)$:

(4) $$n_{i}^{m}\left(r\right)=\sum_{\begin{array}{c} j=1\\ j\neq i \end{array}}^{N-m}\Theta \left(i,j,m,r\right),$$

where:

(5) $$\begin{array}{cc} \Theta (i,j,m,r) & =\left\{\begin{array}{cc} 1: & {\left|\left|{\vec{x}}_{i}-{\vec{x}}_{j}\right|\right|}_{m}\leq r\\ 0: & otherwise. \end{array}\right. \end{array}$$

Similarly, for vectors of length m+1:

(6) $$n_{i}^{m+1}\left(r\right)=\sum_{\begin{array}{c} j=1\\ j\neq i \end{array}}^{N-m}\Theta \left(i,j,m+1,r\right).$$

In Equations (4) and (6), please note that j≠i, meaning that self-matches are excluded (comparison of a vector with itself).

The measures of similarity $B_{i}^{m}\left(r\right)$ and $A_{i}^{m}\left(r\right)$ between templates of length *m* and m+1, respectively, are:

(7) $$\begin{array}{cc} B_{i}^{m}\left(r\right) & =\frac{1}{N-m}n_{i}^{m},\;i=1,2,\cdots ,N-m, \end{array}$$

(8) $$\begin{array}{cc} A_{i}^{m}\left(r\right) & =\frac{1}{N-m}n_{i}^{m+1},\;i=1,2,\cdots ,N-m. \end{array}$$

The mean values of these measures of similarity are computed next:

(9) $$\begin{array}{cc} B^{m}\left(r\right) & =\frac{1}{N-m}\sum_{i=1}^{N-m}B_{i}^{m}\left(r\right), \end{array}$$

(10) $$\begin{array}{cc} A^{m}\left(r\right) & =\frac{1}{N-m}\sum_{i=1}^{N-m}A_{i}^{m}\left(r\right). \end{array}$$

Sample Entropy is given by the formula:

(11) SampEn(m,r)→∞,when A=0=lnBA,otherwise.

## 3. The Straightforward Implementation

In the implementation of the definition presented above (Section 2), we need two variables *A* and *B* to count the total number of similar points and a nested loop to compare all vectors with each other. An algorithm computing Sample Entropy follows (Algorithm 1). This algorithm is based on the definitions, and some basic improvements have been introduced that made the implementation simpler and, at the same time, faster.

**Algorithm 1:** Straightforward01: A=B=0      // initialize similarity counters02: **for  **
i=1…N−m:      // create all pairs of vectors03:   **for  **
j=i+1…N−m:04:     **for  **
k=0…m−1:      // check vectors *i* and *j* in *m*-dimensional space05:       **if  **
$|x_{i+k}-x_{j+k}|$>*r* **then:** **break**06:     **if  **
k=m   **then:  **       // if found to be similar07:      B=B+1       // increase similarity counter08:      **if  **
$|x_{i+m}-x_{j+m}|\;<\;r$   **then:  **       // check for similarity in m+1 dimensional space09:       A=A+1       // increase similarity counter10: A=A(N−m)2; B=B(N−m)2       // counters become probabilities11: **if  **
A=0   **then:  **
SampEn→∞12: **else:  **
SampEn=lnBA       // SampEn is finally computed

The input time series is *x*, *m* is the embedding dimension, and *r* is the threshold distance. The counters *A* and *B* (line 01) are initialized to zero. Then, all possible pairs of vectors are checked for similarity (lines 02–03). The index *j* of the second *for* ranges from i+1 to N−m to avoid unnecessary double checks: it is not necessary to check pair $\left(\vec{x_{j}},\vec{x_{i}}\right)$ when pair $\left(\vec{x_{i}},\vec{x_{j}}\right)$ has already been checked. Additionally, self matching checks are avoided; i.e., vector $\vec{x_{i}}$ with vector $\vec{x_{i}}$. In lines 04–05, each pair of vectors in the *m*-dimensional space is checked for similarity. Please note that in the similarity check, not all *m* comparisons between the elements are necessary. If one comparison fails, then the similarity test stops immediately, exiting the loop. If the vectors are found to be similar (line 06), the counter *B* is increased (line 07). Then, the similarity check is performed for the corresponding vectors in the (m+1)-dimensional space. Since the similarity check for the *m* first elements has already succeeded (lines 04–06), only the last elements of the two vectors need to be checked (line 08). In case of success, *A* is increased (line 09). Next, the probability of two vectors being similar in the *m* and m+1 dimensional space is computed (line 10), even though this is not necessary, since the two denominators will be simplified in division in the next step. Finally (lines 11–12), SampEn is returned as the logarithm of the ratio of *A* and *B*, when A≠0. Otherwise, it is infinite.

Some implementation details: It is important to note that the code was optimized after several tests. The use of *break* in C was proved to be the optimal solution, significantly affecting the overall execution time. The same technique was selected for all algorithms, when this was possible.

## 4. Computation Using *kd*-Trees

A *kd-tree* is a binary tree, each node of which is a vector. The tree is organized like a binary tree. However, when transversing it, we decide if we have to move towards the left or the right child by comparing the $k_{th}$ element of the vector we are looking for, with the $k_{th}$ element of the vector stored in the node. The value of *k* is computed from the level of the visited node: k=lvmodm, where lv is the level of the visited node (the level of the root is considered as 0) and *m* is the size of the vector.

In our problem, the purpose of transversing is not to locate a specific node, but all nodes which are similar to the given vector. We call this kind of searching *range search*. In range search it might be necessary to visit both children, according to the value of *r*. For example, if the vector we are looking for is (3,5,6), the vector in the node is (3,4,6), r=2 and lv=1, then we compare the second elements of the vectors (i.e., 5 and 6) and we decide to move towards the right child. However, since r=2, nodes with their second element equal to 3 are also candidates for being similar and are located under the left child. Thus, in this example we have to visit both left and right children.

The algorithms [20,21] have been proposed for fast computation of Sample Entropy using kd-trees. They first construct the kd-tree and then use range search for finding the similar vectors. Proposed here is an algorithm which searches for similar points, before the final kd-tree is constructed. This is in accordance with the definition of Sample Entropy which avoids self matches. It is also a trick to avoid the comparison between the pair of vectors $\vec{x_{j}}$ and $\vec{x_{i}}$, when the pair of vectors $\vec{x_{i}}$ and $\vec{x_{j}}$ has already been tested for similarity in a previous step. This improvement makes the algorithm two times faster, compared to the descriptions given in [20,21]. The pseudocode follows (Algorithm 2):

**Algorithm 2:**
*kd*-Tree Based01: A=B=0               // initializations02: kd=
**empty  **03: **for  **
i=1…N−m:                 // for every vector04:  $t_{A},t_{B}=range\_search\left(kd,i\right)$               // count the similar vectors already in the tree05:  $A=A+t_{A}$
**;  **$B=B+t_{B}$               // update the similarity counters06:  insertkd(kd,i)               // and then insert the vector in the tree07: **if  **
A=0   **then:  **
SampEn→∞08: **else:  **
SampEn=lnBA               // SampEn is finally computed  


The algorithm is simple. Similarity counters *A* and *B* are initialized to zero (line 01) and the kd-tree to empty (line 02). Next, for every vector which is to be inserted in the kd-tree (line 03), we first perform range search to find the similar vectors already in the tree (line 04), we update the similarity counters *A* and *B* (line 05), and then we insert the vector in the tree (line 06). Sample Entropy is computed at lines 07 and 08.

The algorithm is simple. Similarity counters *A* and *B* are initialized to zero (line 01) and the kd-tree to empty (line 02). Next, for every vector which is to be inserted in the kd-tree (line 03), we first perform range search to find the similar vectors already in the tree (line 04), we update the similarity counters *A* and *B* (line 05), and then we insert the vector in the tree (line 06). Sample Entropy is computed at lines 07 and 08.

Some implementation details: Recursive functions were not used in order to avoid function call delays. Instead, a stack was implemented, again without the use of functions for the stack operations. For the kd-trees representation, three integer arrays were used, each of size *N*. The first had the indexes of the vector in the time series, the second the indexes of the left children, and the third the indexes of the right children, avoiding delays due to structure complexity, pointer handling, and dynamic memory allocation for each tree node.

## 5. The Bucket-Assisted Algorithm

The bucket-assisted algorithm is an extension of the algorithm published in [19] for the computation of Approximate Entropy. The algorithm has been adapted to the definition of Sample Entropy and also improved to present even faster execution times. These two modifications speed up the algorithm remarkably, and will be described in this section.

In [19], we proposed a fast algorithm for computing ApEn. In that algorithm, we used a series of buckets, and we put the candidate points to be similar to each other in neighboring buckets.

The main idea was to integrate the given series *x* and create a new series *X* such that:

(12) $$X=X_{1},X_{2},\cdots ,X_{N-m+1}$$

where:

(13) $$X_{i}=x_{i}+x_{i+1}+\cdots +x_{i+m-1}.$$

Consider a set of buckets:

(14) $$B=\{B_{1},B_{2},\cdots ,B_{h_{N}}\},$$

which consists of $h_{N}$ buckets of equal size *r*, where

(15) $$h_{N}=\left\lceil X_{max}/r\right\rceil .$$

Now, point $X_{i}$ is mapped into bucket $B_{h}$ when $h=\lceil X_{i}/r\rceil$. When a point $X_{i}$, which corresponds to the vector ${\vec{x}}_{i}$, is mapped into the bucket $B_{h}$, then all points similar to $X_{i}$ are mapped into one of the buckets: $B_{h-m},B_{h-m+1},\cdots ,B_{h},B_{h+1},\cdots ,B_{h+m}$. Please see [19] for the proof.

**Figure 1:**
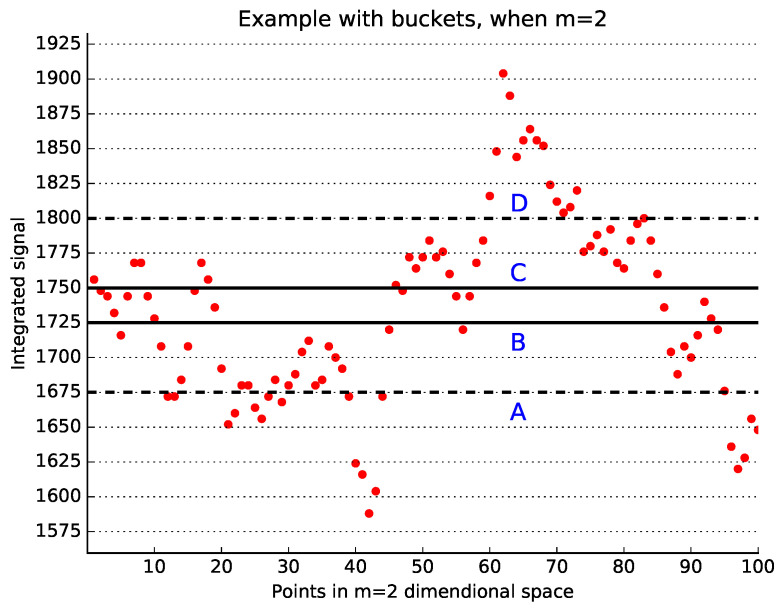
Example of the bucket-assisted algorithm. The integrated signal is depicted here. Points between the solid lines *B* and *C* can be similar only to those points laying between the dashed lines *A* and *D*. However, it is sufficient to check for similarity only in those points located between lines B−C and A−B.

A graphical explanation of the main idea of the bucket-assisted algorithm is shown in Figure 1, where m=2 and the bucket size is 10 ms. Vectors in the bucket BC (solid lines) can be similar only to the vectors between lines *A* and *D* (dashed lines). However, it is not necessary to examine both pairs $\left({\vec{x}}_{i},{\vec{x}}_{j}\right)$ and $\left({\vec{x}}_{j},{\vec{x}}_{i}\right)$ for similarity, as discussed above. Thus, the vectors in the bucket BC are checked for similarity only with those vectors located between lines *A* and *B*, and then between lines *B* and *C*.

One of the main contributions of this work is an extension to the bucket-assisted algorithm, which further speeds up the execution time. The modifications are the following:

- points in the buckets are sorted according to the first element of the vector
- buckets are divided again into smaller buckets (of size smaller than *r*).

These two modifications are enough for a significant speedup, as shown later in Section 7. Please remember that the similarity test fails if the absolute value of at least one of the differences between the corresponding elements of the examined vectors is larger than *r*; i.e.,:

$${\left|\left|{\vec{x}}_{i}-{\vec{x}}_{j}\right|\right|}_{m}\leq r$$

(16) $$\Leftrightarrow \left|x_{i}-x_{j}\right|\leq r,\left|x_{i+1}-x_{j+1}\right|\leq r,\cdots ,\left|x_{i+m-1}-x_{j+m-1}\right|\leq r\;.$$

Thus, a reasonable approach would be to have the points in the buckets sorted according to the first element of the vector, perform the comparison $|x_{i}-x_{j}|\leq r$, and exclude the points that failed this test from the following comparisons. A low overhead binary search O(nlogn) algorithm could be used. Then, the points could be sorted again based on the second element of the vector and perform the comparison $|x_{i+1}-x_{j+1}|\leq r$ until the last comparison $|x_{i+m-1}-x_{j+m-1}|\leq r$ is reached. The points that pass these tests can be considered as similar. However, this approach requires more sorting, since for every examined point we have to sort up to m−1 times. This is relatively expensive, even when we use a low overhead sorting algorithm such as quick sort O(nlogn). Thus, the approach we selected was to sort the points in the buckets only once and excluded from further comparisons only those points that failed the first of the above tests; i.e., $|x_{i}-x_{j}|\leq r$. The algorithm continues by performing the rest of the comparisons of Equation (16) by testing the corresponding elements of each pair of vectors. It can be proved experimentally that the proposed modification adds a significant speedup to the execution time of the algorithm.

**Figure 2:**
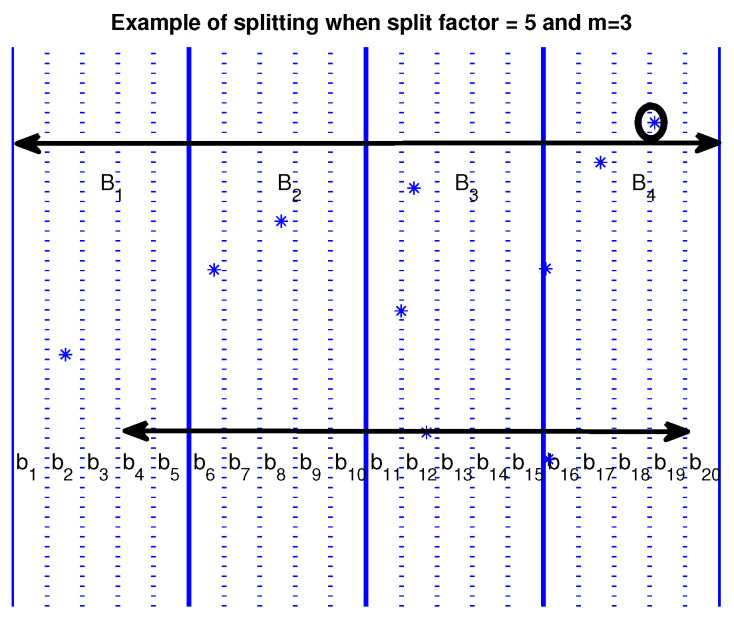
Splitting large buckets into smaller ones. Asterisks are points of the integrated signal. This splitting of the buckets into smaller ones can lead to an increased number of avoided comparisons. For example, for the point marked by the small circle belonging to the bucket b19, comparisons are reduced by approximately 20%.

Some more speedup (also significant) can be achieved by dividing the large buckets into smaller ones. A finer-grained distribution of the points can be achieved by dividing large buckets into smaller ones, avoiding even more comparisons, as shown in Figure 2. When using the large buckets $B_{1},B_{2},B_{3},B_{4}$ for m=3, the point marked by a small circle belongs to bucket $B_{4}$, must be compared for similarity with every other point in buckets $B_{1},B_{2},B_{3},B_{4}$. When using the smaller buckets $b_{1},b_{2},\cdots ,b_{20}$, the same point belonging in bucket $b_{19}$ need to be compared only with points in buckets $b_{4},b_{5},\cdots ,b_{19}$. With this refinement, we can exclude a considerable number of smaller buckets (4 out of 20 in our example) from the comparisons. The number by which a larger bucket is split into smaller ones is a parameter for the problem. We will call it the *split* factor and symbolize it as $r_{split}$. The number of smaller buckets that can be excluded from the comparisons is determined by $1/r_{split}$ of the total number of the smaller buckets.

The algorithm in a detailed description in pseudocode follows (Algorithm 3). Again, *x* is the input signal, *m* the embedding dimension, and *r* the threshold distance.

**Algorithm 3:** Bucket-Assisted01: **for  **i=1…N−m:   $X_{i}=\sum_{k=0}^{m-1}x_{i+k}$               // integrated signal02: $X_{min}=\min\left(X_{i}\right)$03: **for  **
i=1…N−m:   $X_{i}=X_{i}-X_{min}+1$               // normalization04: $N_{b}=\left\lceil max\left(X_{i}\right)/r/r_{split}\right\rceil$               // number of buckets05: **for  **
i=1…N−m:   $bucket_{b}=\mathrm{empty}\;$06: **for  **
i=1…N−m:                 // fill in the buckets07:     $b=\lceil X_{i}/r/r_{split}\rceil$08:     $bucket_{b}=bucket_{b}\;\cup \;\left\{\;\vec{x_{i}}\;\right\}$09: **for  **
b=1…N−m:                 // sort vectors according to first element10:     $b_{ordered}=\left\{\;\vec{x_{i}}\in bucket_{b}:x_{i}\leq x_{i+1}\;\right\}$11:     $bucket_{b}=b_{ordered}$12: A=B=013: **for  **
$i_{b}=1\ldots N_{b}$:                 // for every bucket14:     **for  **
$j_{b}=i_{b}-m\cdot r_{split}\ldots i_{b}-1,\;j_{b}0$:                 // visit all buckets possibly containing similar vectors15:         **for  **$\vec{x_{i}}$, $\vec{x_{i}}\;\in \;bucket_{i_{b}}$:  16:             $candidates=\{\;\vec{x_{j}}\in bucket_{i_{b}}:\;x_{j}-r\leq x_{i}\leq x_{j}+r,\;ij\;\}\;\;\;\cup$
17:                 $\cup \;\{\;\vec{x_{j}}\in bucket_{j_{b}}:\;x_{j}-r\leq x_{i}\leq x_{j}+r\;\}$      // exploit sorting to exclude some comparisons18:             **for  **$\vec{x_{j}}$, $\vec{x_{j}}\;\in \;candidates$:  19:                 **if  **
$\left|\right|\vec{x_{i}}-\vec{x_{j}}{\left|\right|}_{m}\leq r$   **then:  **               // similarity check20:                     B=B+121:                     **if  **
$|x_{i+m}-x_{j+m}|\leq r$   **then:  **
A=A+122: **if  **
A=0   **then:  **
SampEn→∞23: **else:  **
SampEn=lnBA               // SampEn is finally computed

A less formal description of the algorithm follows. We first integrate the signal using a window of size *m* (line 01). The integrated signal *X* is normalized so that $min(X_{i})=1$ (lines 02–03). The number of buckets is equal to the maximum value of the integrated signal divided by the threshold distance *r* and by the split factor $r_{split}$ (line 04). Next (line 05), we initialize the set of buckets bucket to the empty set. To fill the buckets, we select the appropriate bucket for each vector $\vec{x_{i}}$ (line 07) and we add it in this bucket (line 08). Next, we sort the vectors in each bucket according to their first element (lines 09–11).

For the similarity check, we need two counters. We use *B* for the *m* dimensional space and *A* for the m+1 dimensional space. These two counters are initialized to zero (line 12). For every bucket $i_{b}$ (line 13), we check for similar points in all $j_{b}$ buckets in which similar points are possible to be found (line 14). For every point in the examined bucket $i_{b}$ (line 15), we find all points that are not excluded from similarity due to the distance of their first elements (lines 16–17). Since points are sorted according to their first element, this procedure is rapid with complexity only O(logn). In the next step, we check for similarity all pairs of candidate points (lines 18–21) with the same method as it was described in the simple algorithm. SampEn is computed at the two last lines of the pseudocode (lines 22–23).

## 6. A Lightweight Algorithm

Typically, values of the parameter *m* which are used for SampEn estimations are m=1,⋯,3 [12,14,22]. However, recently in [23,24] it was recommended that m=1 in short time series keeps the variation smaller and improves the confidence of the estimates of entropy. Here, we propose an algorithm for fast computation of Sample Entropy which is straightforward when m=1. However, the algorithm is also fast for small signal lengths and other values of *m*. Since it has a simple implementation, we will call it a *lightweight* algorithm.

The algorithm reduces the number of comparisons between points by sorting the original series $x_{i}$. For this purpose, a fast sorting algorithm is used with O(nlogn) complexity. Then, we consider only those sequences for which the first elements are within the allowed tolerance: ${\vec{x}}_{i}$ and ${\vec{x}}_{j}$, i.e., $x_{j}\leq x_{i}+r$. Since the original series is sorted, it is not necessary to consider those cases for which $x_{j}\geq x_{i}-r$, as they were already included. The pseudocode follows (Algorithm 4):

**Algorithm 4:** Lightweight01: $ord_{x}=\left\{\;x_{i}:\;x_{i}\leq x_{i+1}\;\right\}$      // sort x in ascending order02: $pos_{x}=\left\{\;i:\;ord_{x_{i}}=x_{pos_{x_{i}}}\;\right\}$       // remember original positions03: A=B=004: **for**
i=1…N−m:05:  $candidates_{i}=\left\{\;ord_{x_{j}}:\;ord_{x_{j}}\leq ord_{x_{i}}+r\;\right\}$ // points of the ordered series matching other points within *r*06:  $a=pos_{x_{i}}$07:  **for**
$ord_{x_{j}}\in candidates_{i}$:08:   $b=pos_{x_{j}}$09:   **if** $\left|\right|\vec{x_{a}}-\vec{x_{b}}{\left|\right|}_{m}\leq r$ **then:**       // similarity check10:    B=B+111:    **if** $|x_{a+m}-x_{b+m}|\leq r$ **then:** A=A+112: **if** A=0 **then:** SampEn→∞13: **else:** SampEn=lnBA      // SampEn is finally computed

In the lightweight algorithm, the series is sorted (line 01) and the original positions of the elements are stored for later reference (line 02). Similarity counters *A* and *B* are initialized to zero (line 03). Then, for any sample $x_{i}$ of the ordered series, starting from its beginning, all those other samples $x_{j}$ such that $x_{j}\leq x_{i}+r$ are included in the list of possible candidate matches (lines 04–05). The search for candidates is performed on the sorted series, with a binary search, which at worst is O(logn). The stored positions $pos_{x}$ are used to locate the original locations *a* and *b* for $x_{i}$ and any of the $x_{j}$ elements in the candidates list, respectively (lines 06–08). The vectors ${\vec{x}}_{a}$, ${\vec{x}}_{b}$ of length *m* starting at *a* and *b* are checked, and if ${\left|\left|{\vec{x}}_{a}-{\vec{x}}_{b}\right|\right|}_{m}\leq r$, the counter *B* is incremented. If the two further elements at positions a+m and b+m are closer than the threshold *r*, the counter *A* is also incremented (lines 09–11). Sample Entropy is finally computed at lines 12 and 13.

## 7. Experimental Results

In order to evaluate/compare the four algorithms, we performed four experiments with two different datasets. Both datasets are publicly available from Physionet [25]. The first one consists of 24 h of recordings of healthy subjects in normal sinus rhythm (nsr2 dataset). The second one consists of 24 h of recordings of congestive heart failure patients (chf2 dataset). For both datasets, the original electrocardiogram (ECG) recordings were digitized at 128 samples per second, and the beat annotations were obtained by automated analysis with manual review and correction.

The two datasets present different signal characteristics. As expected, the mean value of the chf2 dataset is lower than that of nsr2. Due to the large number of ectopic beats, the chf2 dataset presents larger standard deviation. The existence of ectopic beats influences both the mean value and the standard deviation of the signals. Thus, we removed the ectopic beats (a common practice in HRV analysis) and created two more datasets with different characteristics. The resulting four datasets were the basis for our comparisons. We will refer to them as nsr2, chf2, $nsr2_{f}$, and $chf2_{f}$, where the index *f* comes from the word *filtered*. Average values for the mean and the standard deviation of each dataset are presented in Table 1. It is not a surprise that the standard deviation of the $chf2_{f}$ dataset is the lowest of all, since it reflects the reduction of the complexity of the heart as a system, due to the heart failure disease.

Experiments with all four algorithms were conducted on a 4-core desktop computer (3.6 GHz Intel Xeon E5-1620 processor; 16 GB of RAM; Linux OpenSuse 42.2 x86_64 OS). Code was optimized as much as possible for all algorithms, and the parameter −O3 was used in the GNU Compiler Collection (GCC 4.8.5).

Ten signals were randomly selected from each dataset. The mean execution time for each algorithm and each dataset was computed. Each experiment was performed 100 times, thus the reported execution time is the mean value of 1000 runs.

In order to exclude overheads from the computation time, we first read all input data and stored them into matrices. Then, inside the outer loop (which repeats the experiment 100 times), and before the inner loop (which computes Sample Entropy for the ten signals), we started the timer by using the C function call clock_tclock(void). We used the same call after the inner loop and accumulated all time intervals of all 100 repetitions to estimate the total and then the execution time.

**Figure 3:**
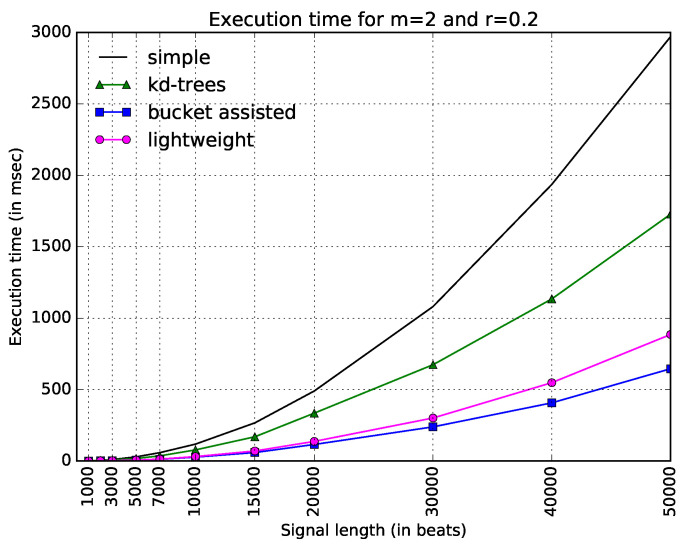
Execution time of all algorithms for the typical values of parameters *m* and *r* (m=2 and r=0.2) and various signal lengths (*N*).

We will start with the experimental result collected from the nsr2 dataset, and then we will discuss differences observed in the other datasets. Figure 3 shows execution times for all four algorithms and the typical values m=2 and r=0.2. The straightforward implementation is the slowest of all, becoming especially slow for large values of *N*. The improved version of kd-trees—as described earlier in this paper—is faster, but not as fast as the other two algorithms. In this figure, the bucket-assisted seems to present the best results, followed by the lightweight algorithm.

The parameter $r_{split}$ for the bucket-assisted algorithm was selected to be equal to 5. We did not try to completely optimize it. We ran the code for several inputs and the typical parameters m=2 and r=0.2, and selected a good and easy-to-remember value of $r_{split}$ for them. Since we did not want to be less fair to the rest of the algorithms and not optimize the results of the bucket-assisted algorithm with an additional parameter, we kept the value $r_{split}=5$ the same for the rest of our experiments. However, we also did some sensitivity analysis on the value of $r_{split}$, which will be discussed at the end of this section.

**Figure 4:**
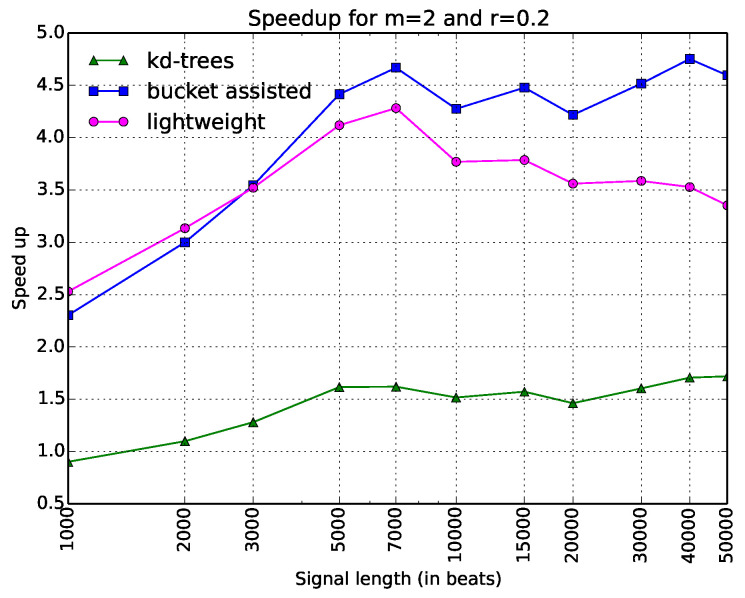
Execution time of all algorithms as a speedup gained from the simple one. Typical values of parameters *m* and *r* (m=2 and r=0.2) have been selected. The *x*-axis is in a logarithmic scale.

One can note that in Figure 3, it is difficult to see the behavior of the algorithms for low values of *N*. For this reason, we added another figure, which gives the same information in a different way. In Figure 4, the *x*-axis is in logarithmic scale. The values in the *y*-axis do not express execution time, but speedup, dividing the execution time of each algorithm with respect to the straightforward one, which we considered as a reference. Expressing the results in terms of a well-defined algorithm—also implemented in a standard programming language, which introduces minimal overhead—allows other researchers to compare their results easily with the ones given in this paper.

Thus, there are only three curves in this figure. The information is depicted in a clearer way. Here, one can see that the bucket-assisted algorithm outperforms the other algorithms for values of *N* approximately larger than 3000 beats. For signals smaller than 3000 beats, the lightweight algorithm gives the lowest execution times.

**Figure 5:**
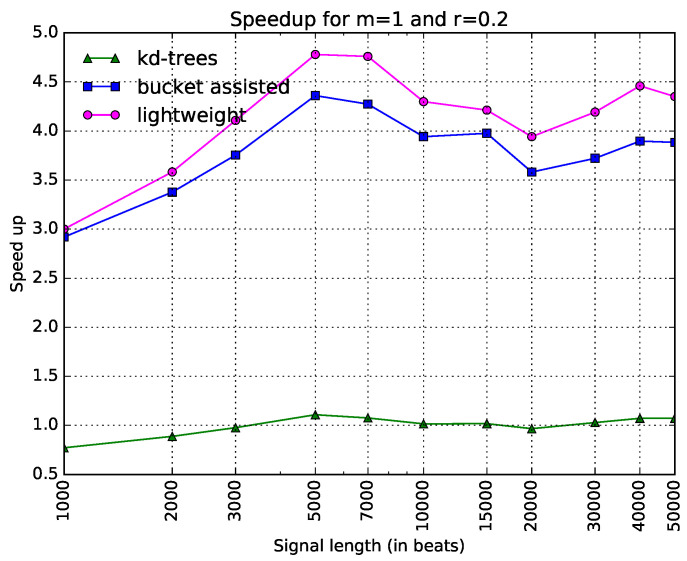
Execution time of all algorithms as a speedup gained from the simple one, when m=1 and r=0.2. The *x*-axis is in a logarithmic scale.

**Figure 6:**
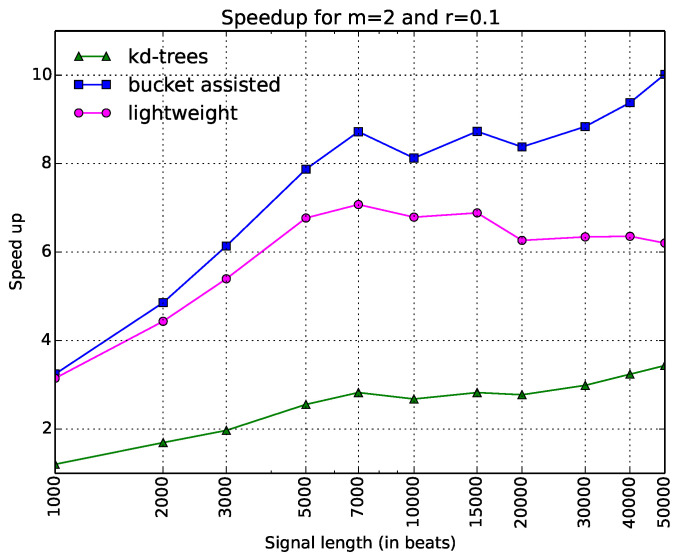
Execution time of all algorithms as a speedup gained from the simple one, when m=2 and r=0.1. The *x*-axis is in a logarithmic scale.

Since this kind of diagram seems more illustrative than the one in Figure 3, we will present the rest of the diagrams in the same way. In Figure 5, speedups for the parameters m=1 and r=0.2 are shown. One can note here that the lightweight algorithm is always faster. The kd-tree algorithm presents poor results. In Figure 6, the speedups when m=2 and r=0.1 are presented. Here, the bucket-assisted algorithm is always faster. The kd-tree algorithm again presents poorer results.

The experiments with the three other datasets gave similar but interesting results, since they helped us to make some additional conclusions. In all cases, the kd-tree algorithm was slower than both the bucket-assisted and the lightweight algorithm. We will continue the comparison between the two latter.

For large values of *N* (N>10,000) the bucket-assisted algorithm was always faster than the lightweight one. As the value of *N* decreases, the lightweight algorithm presents lower execution times.

The removal of ectopic beats gave an advantage to the bucket-assisted algorithm. The lightweight algorithm gave its best execution times for the chf2 dataset and then for the nsr2 dataset—the two datasets with the higher variability. The removal of ectopic beats decreased this variability, and for the same values of *m*, *r*, and *N*, the bucket-assisted significantly improved its performance, almost always presenting better results with the $chf2_{f}$ dataset (the one with the smallest variability).

The relation of the input parameters, the characteristics of the input signals, and the performance of the algorithms is difficult to predict or model. Some general conclusions can be made, but it is certain that each algorithm has a different reason to be used. To give a more detailed image of the relation of the input parameters, the characteristics of the signals, and the execution times, we added a table presenting—for each value of *m*, *r*, and *N*—the number of datasets for which each algorithm performed better. In Table 2, the first number is the number of datasets for which the bucket-assisted algorithm was faster, while the second one is the number of datasets for which the lightweight algorithm was faster. The table depicts only values of N≤10,000.

A last issue to discuss is the question of which value should be selected for the $r_{split}$ factor. We chose 5 for the reasons we mentioned earlier; however, this value is not necessarily the optimal one. In order to perform a sensitivity analysis for the $r_{split}$ factor, we selected the typical values used for Sample Entropy for the parameters *m* and *r* (m=2, r=0.2). We ran the algorithm for different values of $r_{split}$ factor and for different values of *N*. The optimal value of the $r_{split}$ factor was selected for each *N*. We noticed that the larger the value of *N*, the larger the value of $r_{split}$ factor that gave the optimal results. For N<3000, the best values ranged from 2 to 5. For N20,000, the optimal value was close to 15. Despite the small values of *N*, the selection of the $r_{split}$ factor was not crucial, since there was a plateau of values which presented similar execution times. If we try to explain this behavior, a large number of samples would lead to overcrowded buckets. By splitting the buckets into smaller ones, we can achieve a much better distribution, which leads to better execution times.

## 8. Discussion of Related Work

To the best of the authors’ knowledge, the first algorithm for fast computation of ApEn was published in [26]. This algorithm is of $O(N^{2})$ complexity, does not avoid comparisons, and also has a $O(N^{2})$ spatial complexity, even though it can be implemented with a spatial complexity of O(N).

Another algorithm for SampEn is available in [25]. The algorithm builds up templates matching within the tolerance *r* until no match is found, and keeps track of template matches in counters $A_{k}$ and $B_{k}$ for all lengths *k* up to *m*. Once all the matches are counted, Sample Entropy is computed. This algorithm has been designed to compute Sample Entropy for all values of *m* at once. Thus, a straight comparison with the proposed algorithms may not be fair, since the target of the algorithms is different. However, the core of the algorithm is similar to the straightforward implementation we described. Additionally, the modification of the proposed algorithms to target the computation of all values of *m* is possible, but is not an aim of this paper.

In [20], apart from the algorithm for kd-trees, they also presented an algorithm for computing Approximate and Sample Entropy for signals whose elements belong in a definite set of values. It is also based on kd-trees, and exploits the fact that the height of the tree can be limited, and that more than one vector can be stored in the tree node.

The authors of [21] made a theoretical study of the problem which leads to a lower complexity algorithm again based on the kd-trees, which might perform better in very long signals. However, as in [20], with the size of the signal we used, the overhead for constructing the kd-tree and the overhead introduced for a single comparison led to much larger execution times than those obtained with the bucket-assisted or the lightweight algorithms. The same conclusion was drawn in a paper which proposed a fast algorithm for fractal dimension estimation [27]—a similar problem with the one studied here. This paper proposed an algorithm checking for neighboring points in an *m*-dimensional space by separating the *m*-space into orthogonal subspaces and mapping *m*-dimensional points onto these subspaces. It also compared this approach with another one, published before, which used kd-trees for the same purpose [28] and concluded that the algorithm with the subspaces was faster. The approach with the buckets reduces the complexity of handling *m*-dimensional spaces, since handling *m*-dimensional subspaces requires a large amount of memory or alternatively significant overhead to map the *m*-dimensional subspaces onto simpler structures and then manage these structures.

## 9. Conclusions

In this paper, three Sample Entropy computation algorithms were compared with each other, and with an algorithm resulting directly from the definition of the method, in order to decide which one is the fastest (and for which input parameters). The first algorithm was a modified version of an existing one, based on kd-trees. The second one is an extension of another algorithm (the bucket-assisted one), initially proposed for Approximate Entropy, but customized for Sample Entropy and extended to provide even smaller execution times. The last one is a completely new algorithm, which we call *lightweight* since it is “light-weight” compared to the kd-tree-based and the bucket-assisted one. Despite the fact that it was improved, the kd-tree algorithm showed worse execution times than the bucket-assisted and the lightweight algorithms. The lightweight one gave better execution times for specific values of *m* and *r*, and for smaller values of *N*. Thus, the bucket-assisted algorithm and the lightweight one act complementarily, and the one of choice must be selected according to the problem at hand.

## Figures and Tables

**Table 1:** Signal characteristics of the examined datasets.

	nsr2	chf2	$\mathrm{nsr}2_{f}$	$\mathrm{chf}2_{f}$
mean	809 msec	681 msec	807 msec	667 msec
standard deviation	204 msec	369 msec	156 msec	45 msec

**Table 2:** Comparison of bucket-assisted and lightweight algorithms.

	m=1			m=2			m=3		
	r=0.1	r=0.2	r=0.3	r=0.1	r=0.2	r=0.3	r=0.1	r=0.2	r=0.3
N=1000	3/1	2/2	1/3	3/1	3/1	1/3	2/2	1/3	1/3
N=2000	3/1	1/3	1/3	3/1	3/1	1/3	2/2	1/3	1/3
N=3000	3/1	1/3	1/3	4/-	3/1	2/2	4/-	4/-	1/3
N=5000	3/1	1/3	2/2	4/-	3/1	2/2	4/-	4/-	4/-
N=7000	3/1	1/3	2/2	4/-	3/1	2/2	4/-	4/-	4/-
N=10,000	3/1	2/2	3/1	4/-	3/1	3/1	4/-	4/-	4/-
